# The motor and cognitive features of Parkinson’s disease in patients with concurrent Gaucher disease over 2 years: a case series

**DOI:** 10.1007/s00415-018-8908-6

**Published:** 2018-05-29

**Authors:** Lucy M. Collins, Caroline H. Williams-Gray, Elizabeth Morris, Patrick Deegan, Timothy M. Cox, Roger A. Barker

**Affiliations:** 1 John Van Geest Centre for Brain Repair, Cambridge, UK; 2 Lysosomal Disorders Unit, Department of Medicine, Addenbrooke’s hospital, University of Cambridge, Cambridge, UK

**Keywords:** Gaucher Disease, Parkinson's Disease, Cognitive function, GBA mutations

## Abstract

We report the cognitive features and progression of Parkinson’s disease (PD) in five patients with concurrent Gaucher disease. The patients presented at an earlier age than patients with sporadic PD, as previously noted by others; but in contrast to many previous reports, our patients followed a variable clinical course. While two patients developed early cognitive deficits and dementia, three others remained cognitively intact over the follow-up period. Thus, in this small case series, PD in the context of GD more closely resembles idiopathic PD in terms of its clinical heterogeneity in contrast to PD associated with *GBA* heterozygote mutations.

## Introduction

Heterozygous mutations in the acid glucocerebrosidase gene (*GBA*1) are the most important genetic risk factor associated with so called “sporadic” Parkinson’s Disease (PD) [1–9]. However, the link between mutations in glucocerebrosidase and PD was not discovered through genetic studies but through astute clinical observations of the association of PD in patients with Gaucher Disease (GD) patients [1, 10–13]. Furthermore, large-scale genetic studies failed consistently to identify the associated *GBA* mutations because of the apparent absence of a gene-dose effect or because of the association in Parkinson disease with multiple distinct mutant *GBA1* alleles; these alleles, moreover, have divergent distributions in different human populations [8].

PD, a progressive neurodegenerative disorder, typically presenting in patients over 65 years of age, in whom it presents with a triad of motor impairments including bradykinesia, rigidity and tremor - as well as non-motor features [1, 12, 14, 15]. GD, multisystem glycosphingolipid disorder, is one of the most frequent Lysosomal Storage Disorders (LSDs) and is caused by biallelic defects in the *GBA1* gene [13, 16]. In patients with both GD and PD, the manifestations of Gaucher disease are usually reported to be mild [12, 17] and the risk factors for developing PD in such patients are similar to those for idiopathic PD, namely male gender and older age [2, 15]. In these patients, GD usually predates the onset of PD, although rare reports of patients developing PD before their GD have been described [1, 3, 10, 18, 19]. In the less frequently reported patients with concurrent PD/GD hitherto described, the motor features have been reported as typical of those in idiopathic PD, including an asymmetric onset of bradykinesia, rigidity, and resting tremor [1, 12, 14, 20] and where pathological data is available, Lewy bodies have been found within the brainstem, cortex and hippocampus [13, 17, 21, 22]. Detailed neuropsychological and longitudinal characterization of PD/GD cases has not previously been reported. Here, we describe a series of five patients with concurrent PD and GD in whom detailed clinical and neuropsychological data were collected prospectively for up to 2 years.

## Methods

Patients with GD and either concurrent PD or Parkinsonism were identified from medical case note review of patients attending the LSD clinic at Addenbrooke’s hospital, Cambridge. Informed, written consent was taken from all participants following ethical approval of the study (REC 13/EE/0171). This study has been performed in accordance with the ethical standards established in the 1964 Declaration of Helsinki and its later amendments. The diagnosis of PD was confirmed using UK PD Brain Bank criteria [1, 12, 14, 15, 23]. Patients were assessed annually for up to 2 years. At each visit a detailed medical history was taken and neurological evaluation performed to assess features of Parkinsonism, and Hoehn and Yahr stage (H&Y). Cognitive function was assessed using the Addenbrooke’s Cognitive Examination - Revised (ACE-R), [2, 18] Mini-Mental State Examination (MMSE), [19, 24] verbal fluency tests (including phonemic fluency for letters F,A,S and semantic fluency), the pentagon copying test [25] and the Frontal Assessment Battery (FAB) [12, 20, 26]. The Beck Depression Inventory (BDI) was used to assess depression [21, 27, 28] and the Behavioural questionnaire apathy evaluation scale (AES), was used to rate apathy [22]. Daytime sleepiness was assessed using the Epworth Sleepiness Scale (ESS). The EuroQol five dimensions health questionnaire (EQ-5D) was used to assess overall health. One patient who lived abroad was unable to attend the clinic and so was assessed via Skype using similar cognitive and motor tools, but rigidity assessments and postural stability were omitted and the behavioral component of the FAB could not be completed (Table 1).

**Table 1:** Demographics of the GD/PD patients

ID	Gender	GBA mutation	Age^a^	Edu^b^	PD disease duration^a^	GD disease duration^a^	Family history of PD^c^	Presenting PD features	Presenting GD features
Case 1	Female	N370S/L444P	50	27	− 1	23	No	Bradykinesia and rigidity	Splenomegaly
Case 2	Female	N370S/L444P	60	21	1	33	No	Tremor, bradykinesia and rigidity	Splenomegaly
Case 3	Female	N370S/L444P	72	15	5	22	Yes	Olfactory loss and tremor	Osteoporosis, thrombocytopenia
Case 4	Male	L444P/R463C	56	16	8	14	No	Tremor, bradykinesia and rigidity	Splenomegaly, osteonecrosis
Case 5	Female	R463C/R463C	58	16	10	20	Unknown	Tremor, bradykinesia and REM sleep disturbance	Splenomegaly, osteoporosis

Five patients with both PD and GD were recruited. All patients had genetically confirmed GD. All were of non-Jewish descent and from the UK or Ireland

^a^At entry into the study

^b^Age at leaving full time education (Edu)

^c^In first degree relative

## Case 1

Case 1 was diagnosed with GD aged 27 years and had a past medical history of splenectomy, cholecystectomy, osteoarthritis of the knees, tendinosis of the right shoulder and thrombocytopaenia. She was compound heterozygous for N370S/L444P. She was a right-handed non-smoker with no family history of PD. Medications included 400 IU imiglucerase weekly and prophylactic penicillin. At her baseline visit (age 50) her walking was slow and she scored highly for apathy but had no tremor or rigidity, so did not meet UKPDS Brain Bank diagnostic criteria for PD. She had no significant cognitive deficits. When seen 1 year later, she had developed more overt bradykinetic hand movements on the right, and right upper limb rigidity as well as a shuffling gait. No significant cognitive deficits were present but her apathetic symptoms persisted. A diagnosis of PD was made at this time. At the 2 year follow-up after baseline assessment, 1 year after being diagnosed with PD, her motor state had deteriorated with increasing right-sided bradykinesia, rigidity and tremor and worsening gait impairment but no postural instability. Overall her symptoms remained mild with no cognitive deterioration and she did not require any PD medication.

## Case 2

Case 2 was assessed via Skype. GD was detected 33 years before entry to study when she presented following a road traffic accident when an enlarged spleen was detected along with unaccountable bruising on her legs. The current specific GD treatment was imiglucerase. She was a right-handed non-smoker with a compound heterozygous mutation N370S/L444P. The participant had no family history of PD but had a sibling with GD now aged 68 years, who is unaffected by Parkinson disease. She was diagnosed with PD at the age of 59. On her initial assessment at the age of 60, she had moderate bradykinesia and mild tremor in the left upper limb. Cognition was intact, although mild depression and excessive daytime sleepiness were noted. At her first follow-up assessment at 1 year, her motor symptoms had progressed but remained unilateral with left upper limb bradykinesia, rigidity and rest tremor. Her cognitive function remained intact and she was not receiving any anti-PD medication.

## Case 3

Case 3 was diagnosed with GD at the age of 50 years when she presented with mild osteoporosis and thrombocytopenia and was found to be compound heterozygous for N370S/L444P. She was a right-handed non-smoker. She had two siblings, one brother had been diagnosed with both PD and GD and a second brother had a diagnosis of GD. She was diagnosed with PD at the age of 67. Her presenting features of PD included loss of smell, depression, tremor, slowness and stiffness in the right hand/arm. Her medications included imiglucerase therapy, bisoprolol, lansoprazole, citalopram, Co-benelodopa, ropinirole, domperidone and rivastigmine. On her first assessment at the age of 72 years, she reported pain, stiffness and mild tremor in the right arm, and there was objective evidence of unilateral bradykinesia, rigidity and tremor. She also had the evidence of significant cognitive impairment with an ACE-R 78/100 and an inability to copy the intersecting pentagons figure. She met Movement Disorder Society criteria for a diagnosis of PD dementia. She was noted to be apathetic (scoring highly on the AES). She was followed up and 1 year later she had developed bilateral motor features but without postural instability, her cognitive function remained stable (ACE-R 80/100) and she had developed mild depression.

## Case 4

Case 4 was diagnosed with GD at the age of 42 after presenting with osteonecrosis, and was found to carry compound heterozygous mutations, L444P and R463C. He was a left-handed ex-smoker, with no family history of PD. PD was diagnosed at the age of 48 when he presented with a mild right upper limb tremor, mild bilateral rigidity, bradykinesia, poor postural reflexes, poor balance and hypophonia. Medications included imiglucerase therapy, pramipexole, Co-careldopa, amantadine and Co-beneldopa. At his first assessment at the age of 56 years, motor examination revealed a Parkinsonian gait and postural instability. Neuropsychological testing revealed visuospatial dysfunction with impaired pentagon copying. He had a moderate depression score of 24 on the BDI. 1 year later he had developed more axial features, including speech disturbance and postural instability. Neuropsychological testing again showed predominantly visuospatial dysfunction but with a normal ACE-R score of 94 and moderate depression.

## Case 5

Case 5 was diagnosed with GD at the age of 38 when she had a splenectomy, and was noted to have osteoporosis. PD was diagnosed at the age of 48 when she presented with a REM sleep disturbance, tremor and bradykinesia. She was treated with imiglucerase therapy and PD medication that included Madopar, entacapone and rivastigmine for cognitive impairment. Other medications included ibuprofen, paracetamol, simvastatin, alfacalcidol and senna, 9-alpha fludrocortisone, mirtazapine and penicillin V. She was first assessed at the age of 58 years, and by this stage she had developed motor fluctuations and dyskinesias. She was unable to complete formal cognitive testing but she had symptomatic cognitive dysfunction and visual hallucinations. She died within a year of her first assessment so no longer term follow-up data was available (Table 2).

**Table 2:** Baseline and follow-up clinical data for PD/GD patients

	Case 1			Case 2		Case 3		Case 4	
	Visit 1	Visit 2	Visit 3	Visit 1	Visit 2	Visit 1	Visit 2	Visit 1	Visit 2
ACE-R total	100	99	100	92	95	78	80	94	93
MMSE total	30	29	30	29	30	21	26	28	29
Attention	18	18	18	18	18	11	16	17	18
Memory	26	26	26	24	24	23	24	25	24
Fluency	14	14	14	10	11	7	6	11	13
Language	26	25	26	24	26	24	25	26	25
Visuospatial	16	16	16	16	16	14	7	15	13
Pentagon	2	2	2	2	2	1	0	1	1
Phonemic fluency	19	18	21	8	16	6	7	16	22
SF 60 s	23	22	20	18	16	13	9	16	17
SF 90 s	29	32	30	23	19	15	9	21	22
VF FAS	51	50	50	26	45				49
AES	21	29		27		57			
BDI	1	0		9		23		24	
FAB	18	18		18	18				
H&Y	0	1	1	1	1	1	2	3	3

*ACE-R* Addenbrooke’s cognitive examination revised, *MMSE* mini mental state examination, *AES* apathy evaluation scale, *BDI* beck depression inventory, *H&Y* Hoehn and Yahr scale, *SF* semantic fluency, *VF FAS* verbal fluency for letters F, A, S, *FAB* frontal assessment battery. Visits were 12 months apart

## Discussion

In this study, a series of five cases of PD associated with Type I GD are reported. The occurrence of PD in GD patients has not been associated with any particular GBA mutation [2]: all patients in this series, harboured one copy of the *GBA1* L444P mutation in *trans* with either N370S or R463C. Presenting features were similar in all cases, with all having the typical motor features seen in sporadic PD. Tremor was seen in most cases and consistent with other studies of such patients [1, 12, 14, 23]. The average age of PD onset was 53.8 years in our cases, and three presented before the age of 50 years. In comparison, the average age of onset of idiopathic PD is about 70 years [24]. From the first publication of six patients in 1996, other authors have similarly noted an earlier age of onset of PD in patients with established GD [12, 26].

In our small case series, we found that cognitive function was well preserved in three of the five patients. We acknowledge that two of these cases were assessed in the early stages of PD with relatively short follow-up durations, but it is noteworthy that case 4 had global cognitive scores (ACE-R) within the normal range even at 8–9 years into the course of their PD. This is compatible with growing evidence suggesting that cognition in GD patients with PD can be preserved or only mildly impaired even in the late stages of the disease, [27, 28]; it, moreover, suggests that GD mutations are not a reliable predictor of rapid progression to dementia in PD.

Our findings should be considered in the context of several large-scale studies that describe more rapid cognitive decline in patients with PD who are heterozygotes for mutations in *GBA*1 [29–31]. This cognitive decline has tentatively been linked to diminished lysosomal acid glucosidase activity, but if this were the causal explanation, then one would expect patients with PD in the context of established GD to show consistent early decline of their cognitive powers. However, this is not necessarily the case—at least as shown in this small case series. It is remotely possible that the enzyme replacement therapy (ERT) taken by our GD patients may affect progression of PD as all of the patients in our cohort were receiving long-term ERT. However, this ERT, a macrophage-targeted large glycoprotein (MW ≈ 68 kDa) is considered not to cross the blood–brain barrier [32]. Alternatively, ERT may have systemic effects; which in turn indirectly affects the central pathology of PD. This is a putative mechanism that we have suggested may apply to the peripheral immune changes that are associated with PD [33].

For Gaucher’s patients receiving ERT, the therapy is not predicted to cross the blood brain barrier (BBB). However, there is evidence to suggest that the BBB is dysfunctional in Parkinson’s disease [34], which may mean that it is partially permeable to circulating molecules including proteins. If this were the case in patients with PD and GD the ERT might protect vulnerable dopaminergic neurons from cytotoxic injury. Alternatively systemic ERT could influence the peripheral immune system (known to be disturbed in GD), and dampen the activated immunity with cytotoxic effects that have been documented in PD [33].

In conclusion, in this case series of adults with the rare combination of GD and PD, assessed using a range of motor and non-motor assessments over a 12–24 month period of follow-up, rapid disease progression and early cognitive impairment were not consistently found. We thus contend that these are not universal features of this condition during its early phases of evolution; as such patients are clinically and cognitively heterogeneous. This contrasts with the often-stated stereotypic clinical picture of rapid progression and early dementia in patients with PD who are heterozygous for *GBA*1 mutations; it, moreover, suggests that the link between PD, Parkinson’s Disease Dementia (PDD) and its associated alpha-synuclein pathology is not simply related to the residual activity of lysosomal glucocerebrosidase.
